# Systemic Fusariosis: A Rare Complication in Children with Acute Lymphoblastic Leukemia

**DOI:** 10.3390/jof6040212

**Published:** 2020-10-09

**Authors:** Giada Biddeci, Daniele Donà, Giulia Geranio, Silvia Spadini, Maria Grazia Petris, Marta Pillon, Alessandra Biffi, Maria Caterina Putti

**Affiliations:** 1 Maternal and Child Health Department, Pediatric Hematology, Oncology and Stem Cell Transplantation Division, Padua University Hospital, 35128 Padua, Italy; giulia.geranio@gmail.com (G.G.); spadini.silvia@gmail.com (S.S.); mgrazia.petris@unipd.it (M.G.P.); marta.pillon@unipd.it (M.P.); alessandra.biffi@unipd.it (A.B.); mariacaterina.putti@unipd.it (M.C.P.); 2 Maternal and Child Health Department, Pediatric Infectious Disease Division, Padua University Hospital, 35128 Padua, Italy; daniele.dona@phd.unipd.it

**Keywords:** leukemia, Fusarium infection, mycosis

## Abstract

*Fusarium* species are ubiquitous pathogens causing opportunistic infections in immunocompromised patients. Clinical presentation depends on a host’s immunity and can be localized or disseminated. Since there are few reports of disseminated fusariosis in children, we described an unusual case of *Fusarium solani* infection in a 9-year-old child with acute lymphoblastic leukemia (ALL). This patient presented a deep wound in the elbow at diagnosis. During the induction phase of chemotherapy, he developed multiple skin lesions and severe pneumonia; *Fusarium solani* was cultured from the skin lesions. He was treated with a high dose of liposomal amphotericin B, followed by voriconazole. Starting from this peculiar case, we collected all patients with acute leukemia affected by *Fusarium* infection, treated in the pediatric Onco-Hematology Division of Padua University Hospital during the last 20 years. We identified another six cases: all these patients were affected by acute myeloid leukemia (AML) and five of them presented a relapsed/refractory disease. Two out of seven patients died because of infection; five patients recovered from infection, but three out of seven died because of leukemia. Skin lesions in immunocompromised patients should rise the suspicion of disseminated fusariosis. Furthermore, considering the emergence of filamentous fungi in immunocompromised patients, we all should be aware of *Fusarium* infection, reminding us that the diagnosis is important to cure the infection.

## 1. Introduction

*Fusarium*, *Aspergillus*, and Zygomicetes (Mucorales) species are the molds that most frequently cause severe, invasive infections in immunocompromised patients. While infections by *Aspergillus* and Zygomicetes fungi are frequently seen in patients undergoing intensive chemotherapy for acute leukemia, *Fusarium* is only rarely reported as causative agent.

*Fusarium* conidia are widely distributed in soil, plants, and organic substrates [1]. More than 100 *Fusarium* species have been identified; opportunistic *Fusarium* species in humans are grouped into seven complexes: *Fusarium solani*, *F. oxysporum*, *F. incarnatum-equiseti*, *F. fujikuroi*, *F. clamydosporum, F. dimerum*, and *F. sporotrichioides* [2,3]. *Fusarium solani* is the most frequent species, followed by *Fusarium oxysporum*, *Fusarium verticilloides*, and *Fusarium moniliforme* [4].

*Fusarium* species cause a broad spectrum of infections, from superficial (keratitis, onychomycosis) to invasive and disseminated (pneumonia, fungemia, severe skin lesions) [3,4].

Innate immunity plays an important role in the defense against molds, as macrophages and neutrophils can destroy fungal hyphae [5]. Invasive fusariosis shares similar features with invasive aspergillosis and other invasive molds, affecting immunocompromised patients with prolonged neutropenia [6]. In addition, cellular immunity, and in particular T lymphocytes, are important in the defense against molds; this explains the recurrence of fungal infection in patients after hematopoietic cell transplant (HCT) [7].

Here, we present the case of a child with acute lymphoblastic leukemia (ALL), who developed disseminated *Fusarium solani* infection during induction chemotherapy. Starting from this unusual case, we retrospectively identified all cases of proven invasive fusariosis in children affected by acute leukemia, observed in the pediatric Onco-Hematology Division of the Padova University Hospital between the years 2000 and 2020.

## 2. Materials and Methods

From 2000 to 2020, 571 patients affected by ALL and 72 patients affected by acute myeloid leukemia (AML) were treated with AIEOP protocols (AIEOP ALL 2000 and AIEOP BFM ALL 2009; AIEOP AML 2002 and AML 2013) in the pediatric Onco-Hematology Division of the Padova University Hospital.

*Fusarium* infection was documented, according to internationally accepted criteria, by mycological evidence of fungus in biological specimens (blood, BAL, biopsy) in 7 of them, including our index case. Disseminated infection was defined when the presence of fungal pathogen in the blood (fungemia) and/or in two non-adjacent sites. *Aspergillus* galactomannan (GM, normal value < 0.5) was used for early diagnosis and monitoring, as a standardized fungal biomarker test. Introduced as infection marker since 2016 was 1,3-beta-D-glucan (BDG, normal value < 60 pg/mL).

Age, state of leukemia, clinical manifestation of Fusarium infection, and radiological findings in our patients were recorded (Table 1).

## 3. Results

### 3.1. Index Case

**Figure 1:**
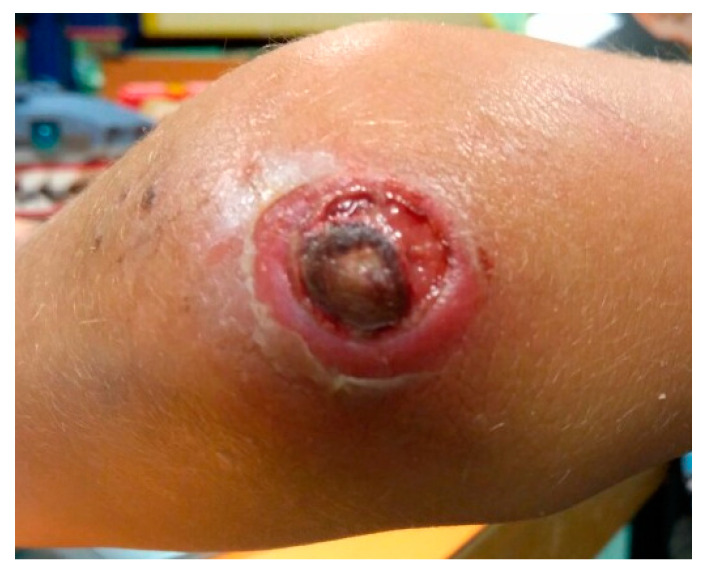
Elbow skin lesion of patient 1. Patient’s parents signed the written inform consent for publication.

**Figure 2:**
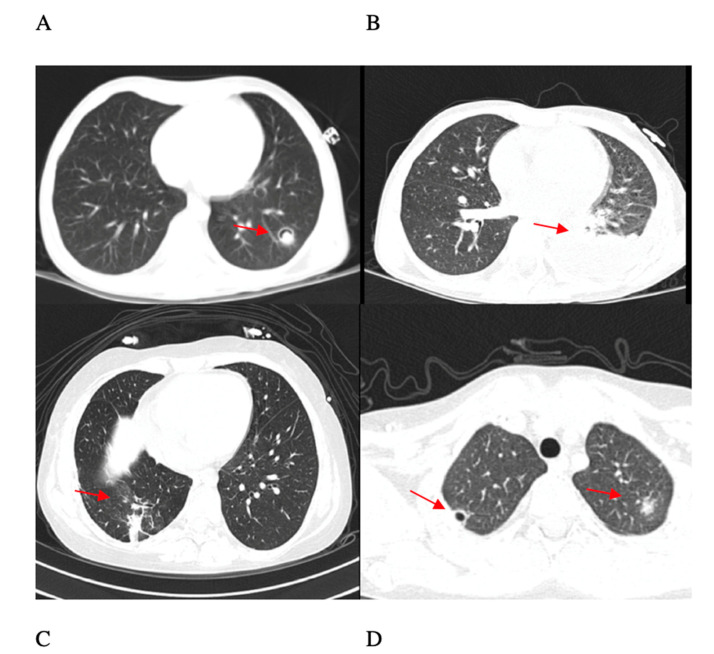
Thoracic CT of patient 1 at day 21 (**A**) and day 35 with initial worsening (**B**); patient 3 (**C**); patient 5 (**D**). Red arrows show the most evident sites of fungal infection.

A 9-year-old child, affected by high-risk ALL, developed *Fusarium* infection during the induction chemotherapy phase. This patient presented with a deep wound in the elbow at the time of ALL diagnosis, at the site of a trauma occurred a few days before (Figure 1). The child presented a deep neutropenia at diagnosis of leukemia (white blood cells 9300/m^3^, neutrophils 50/m^3^). Two weeks after diagnosis, a painful erythematous lesion appeared on the right leg. The lesion presented a liquid core at ultrasound scan, whose drained fluid culture identified *Fusarium solani*. After culture isolation, the strain was identified based on its macroscopic and microscopic characteristic (giant colony and slide culture); molecular study was not performed. The boy suffered from general wasting and profound weight loss, albeit without significant fever. At day 21, he presented cough and a thoracic computed tomography (CT) scan showed multiple nodular lesions and a big cavitation in the left lower lobe (Figure 2). Positron emission tomography magnetic resonance (PET-RMN), performed at day 30, showed another deep lesion in the left gastrocnemius, whose aspiration was also positive for *Fusarium solani*. The GM test was negative, whereas the BDG result was positive (maximum value 350 pg/mL), and it decreased in parallel with the clinical response. He was initially treated with high doses of liposomal amphotericin B, up to 10 mg/kg, with only a partial response in the lung, and then voriconazole was introduced. Therapeutic drug monitoring (TDM) was performed once a week. A daily voriconazole dose of 10 mg/kg was needed to maintain TDM targets of 3–5 mg/L, and it was not associated with toxicity. Antifungal agents were not tested in vitro, so minimum inhibitory concentration (MIC) was not determined.

Thoracic CT presented an initial worsening at day 35, with slow improvement from day 45. Despite this infection, thirty days of induction chemotherapy was completed, obtaining hematological remission; it was then suspended for one month. Though slowly improving, fungal lesions did not resolve at due time for continuation of standard chemotherapy-based regime, so we decided to introduce a bridging immunotherapy cycle with anti CD19/CD3 monoclonal antibody Blinatumomab to control his leukemia and allow recovery from the *Fusarium* infection. Fungal lung lesions resolved under voriconazole therapy within two months. A maintenance therapy with voriconazole was continued, and it is still ongoing. The lesion of the elbow resolved only after 6 months.

### 3.2. Other Patients in the Series

Among all children treated for neoplasms in our clinic since 2000, seven cases of fusariosis were identified. Clinical features of these seven patients are shown in Table 1. Six of them were affected by AML with a prevalence of fusariosis in our AML pediatric population equal to 8.3%. Five of these children had relapsed or refractory disease. Only the case reported above was affected by ALL. No cases of fusariosis were identified among children with solid tumors. The first patient presented a probable infection, because the blood culture was negative, whereas the other six children presented a proven infection with fungemia.

Infection was disseminated in five out of seven fusariosis patients, all with combined lesions in the skin and in the lung. One case had isolated lung disease and one had a sepsis without precise localization. The diagnosis was based on positive blood cultures in six out of seven cases. In three cases, positive cultural bronchial aspirates lung biopsy and skin biopsy were also obtained. In one case, the diagnosis was confirmed on skin lesion cultural testing. *Fusarium* species characterization was available in two patients, and *Fusarium solani* and *F. verticilloides* were identified. GM was positive in two cases and dubious in another one. BDG was not available for six patients [2,3,4,5,6,7]. None of the patients received antifungal agents for prophylaxis. All six patients with AML were treated with combination antifungal therapy (caspofungin 50 mg/m^2^; voriconazole according to TDM). Surgical resection of the lesion was required in one patient (#5) with lung lobectomy. Interestingly, three out of seven patients months before *Fusarium* infection diagnosis presented with a pulmonary infection diagnosed as aspergillar pneumonia, based on GM positivity (two cases) and radiological imaging. Two out of these three patients had undergone lung lobectomy, but cultures and histologic examination did not confirm the *Aspergillus* infection.

Overall, the outcome of fusariosis was unfavorable in two out of seven cases who died because of *Fusarium* infection; moreover, of the five patients who recovered from infection, three died because of leukemia and two patients are still alive.

## 4. Discussion

The reported rarity of *Fusarium* infection in children with acute leukemia is confirmed also in our experience, occurring in seven out of 643 heavily treated acute leukemia patients. Most of them were affected by AML in an advanced phase and were infected during phases of profound aplasia, as reported in the literature [3,4,8]. All patients were at high risk of opportunistic infection because of deep and prolonged neutropenia and active leukemia. Notably, *Fusarium* infection in the index case occurred at the onset of leukemia presentation, starting from a deep elbow wound. *Fusarium* spore are disseminated in the soil, air, and water [6]. In most cases, the principal portal of entry is the airways, followed by skin and mucosal membranes. This patient indeed has a recent trauma with soil contamination of the lesion.

Skin involvement is often the clue to diagnosis, as cutaneous lesions are observed in 85% of patients with disseminated fusariosis and can often represent one of the first signs of infection [9]. They involve any sites, especially the extremities, with different patterns (papules, nodules, necrotic lesions) and can evolve rapidly. Lung involvement is common in disseminated infection (39%), presenting with nodular and cavitary lesions [4]. In our experience, the most frequent sites of infection were the lung and the skin, frequently combined (four out of seven).

The diagnosis is usually made by culture of skin lesions [4], and also in our case series, skin lesion culture tests were diagnostic in 55% of the cases. A peculiar characteristic of disseminated fusariosis that is not typical of *Aspergillus* infections is the frequent finding of positive blood cultures (41%). Indeed, in our series, blood cultures were positive in six out of seven patients.

Suspect aspergillar pneumoniae was reported in the previous history of three of our patients, and in two (number 2,3), GM was positive. At that time, diagnosis of *Aspergillus* pneumonia was not confirmed, even by histology. BDG can be helpful for establishing the diagnosis at an early disease stage and to monitor the response to treatment for invasive mycoses such as *Aspergillus*, *Candida*, and *Fusarium* [10,11,12], as observed in patient 1. GM presents a cross-reactivity with antigens released by a large number of fungi, as reported in the literature [13,14]. False-positive GM test results for patients with Fusarium infection are also reported [15,16].

Reviewing the literature, only sporadic case reports about disseminated *Fusarium* infection in immunocompromised children are described. The most part of cases is reported in patients affected by leukemia with prolonged neutropenia [17,18]. Mortality rate is high, about 75%. The large case series was described by Hassler et al., who presented 10 cases of fusariosis identified in the Global Fugiscope Registry [19]. Arnoni et al. [20] described four cases of invasive fusariosis, three in children with ALL and one in a patient with Wilms’ tumor. All patients were treated with combination therapy (amphotericin B and voriconazole).

The treatment of invasive fusariosis needs an aggressive anti-mycotic therapy. Anti-mycotic agents are seldom tested in vitro, so the MIC is generally not available. Furthermore, there is little information documenting a correlation between MICs and the clinical outcome [3]. There are no treatment guidelines for patients with invasive fusariosis and because of the lack of clinical trials, the optimal strategy remains unclear. Retrospective studies describe the use of voriconazole as first-line therapy, followed by amphotericin B [21]. Data on combination therapy are limited to sporadic reports and little evidence is available to define standard guidelines. In addition to anti-mycotic therapy, surgical intervention is required in some cases to better control the infection [3,22]. In the majority of cases, antifungal therapy alone is sufficient to cure the infection, but in case of extensive lung involvement or in deep tissue infection, where the concentration of drugs is often insufficient, surgical debridement could be helpful. Surgery should be avoided in immunocompromised patients when a demolitive intervention is required, because it could delay the recovery. Surgery is also useful as a diagnostic tool, when blood culture is negative.

Our patients were treated with double anti-mycotic therapy, combining amphotericin B and voriconazole (six out of seven) or sequentially employing the two drugs (one out of seven). Surgical intervention was required in two cases: patient 5 received lung lobectomy and patient 1 needed drainage and multiple curettages of the skin lesions. In the first case, the lung mycosis was extensive and surgical intervention helped to cure faster the infection, in order to restart the chemotherapy. In the second case, drainage of deep tissue lesions were precious for the correct diagnosis (*Fusarium solani*), because blood cultures were negative. Furthermore, the drainage was useful also to cure the infection, because deep tissues are difficult to reach by therapy.

The outcome of fusariosis is generally poor, depending on the disease status of the patient, with the worst outcome after hematopoietic stem cell transplantation (HSCT) or prolonged neutropenia [7]. Two of our patients died because of the infection, another three patients recovered from fusariosis, but they died because of leukemia progression. Patients 1 and 5 recovered, and they are alive without chemotherapy.

In conclusion, we present a small series of this rare infection in severely treated leukemic patients. Prompt diagnosis and aggressive anti-fungal treatment resulted in cure of the infection in the majority of our cases, however ultimate prognosis depends on the status of leukemia.

## Figures and Tables

**Table 1:** Patients with proved diagnosis of *Fusarium* infection.

Patients	Disease	Age/ Gender	Chemotherapy Phase	Sites of Infection	Diagnosis	Time of Diagnosis (Days)	EORT/MSG	Previous Fungal Infection	Antifungal Therapy (Treatment Duration)	Outcome
1	BCP ALL	9 years/ M	First line therapy (ALL Induction)	Skin, lung	*F. solani*: culture of skin lesions GM− BDG+	7	probable	No	Liposomial amphotericin B followed by voriconazole (2 months)	Recovering, on treatment
2	AML (M6)	8 years/ M	2^ HSCT for 2^ relapse	Lung (ARDS)	*F. verticilloides*: blood culture+ bronchial aspirate GM+	3	proven	Yes: Pneumonia After 2^ HSCT	Caspofungin + liposomial amphotericin B Caspofungin + voriconazole (n.a.)	Dead for infection
3	AML (M4)	2 years/ F	Relapse after the 1^ HSCT	Blood, lung, skin	Blood culture, cultures of skin lesions GM+	5	proven	Yes: Pneumonia First line therapy	Caspofungin + voriconazole (3 months)	Recovered, dead for leukemia
4	AML (M7)	9 years/ F	First relapse	Blood, skin	Blood culture GM+/−	5	proven	No	Caspofungin + voriconazole (n.a.)	Dead for infection
5	AML (M6)	8 years/ M	First line AML therapy	Blood, lung, skin	Blood culture, histological analysis on lung lobectomy GM−	7	proven	No	Caspofungin + voriconazole (2 months)	Recovered
6	AML (M4)	17 years/ M	2^ relapse after HSCT	Lung, skin	Blood culture GM−	3	proven	No	Caspofungin + voriconazole (3 months)	Recovered, dead for leukemia
7	AML (M1)	9 years/ F	Extramedullary relapse after HSCT	Blood	Blood culture, culture of central venous line GM−	5	proven	Yes: Pneumonia First line therapy	Caspofungin + voriconazole (2 months)	Recovered, dead for leukemia

EORT/MSG: European Organization for Research and Treatment of Cancer/ Mycoses Study Group; BCP ALL: B cell precursor acute lymphoblastic leukemia; AML: acute myeloid leukemia; HSCT: hematopoietic stem cell transplantation; GM: galactomannan; BDG: beta-D-glucan; F: female; M: male; n.a.: not applicable. 1^ means first time, 2^ means second time. ARDS: acute respiratory distress syndrome.
